# Association Between Severe Periodontitis and Cognitive Decline in Older Adults

**DOI:** 10.3390/life14121589

**Published:** 2024-12-03

**Authors:** Yash Brahmbhatt, Hend Alqaderi, Zahra Chinipardaz

**Affiliations:** 1 Tufts University School of Dental Medicine, Boston, MA 02111, USA; yash.brahmbhatt@tufts.edu; 2 Department of Public Health, Tufts University School of Dental Medicine, Boston, MA 02111, USA; 3 Dasman Diabetes Institute, Kuwait City 15462, Kuwait; 4 Department of Periodontology, Tufts University School of Dental Medicine, Boston, MA 02111, USA; zahra.chinipardaz@tufts.edu

**Keywords:** periodontitis, oral health, cognitive impairment, dementia, alkaline phosphatase

## Abstract

(1) Background: Periodontal disease, a progressive inflammatory condition, disrupts the oral microbiome and releases inflammatory cytokines, leading to systemic issues, including cognitive decline. This study investigates the association between severe periodontitis and cognitive decline, exploring the role of alkaline phosphatase (ALP), an enzyme linked to systemic inflammation, as an effect modifier. (2) Methods: We analyzed cross-sectional data from the 2013–2014 National Health and Nutrition Examination Survey (NHANES). Severe periodontitis was defined using the Centers for Disease Control and Prevention (CDC) and the American Academy of Pediatrics (AAP) case definition. A weighted multivariable logistic regression model assessed the association between severe periodontitis and cognitive decline. An interaction term examined ALP’s role as an effect modifier. (3) Results: This study included 1265 participants aged 65 and older. After adjusting for confounders, each one-point increase in cognitive function score was associated with a 2% decrease in the odds of severe periodontitis (OR = 0.98; 95% CI = 0.97–0.99; *p* = 0.008). ALP was a significant effect modifier in the relationship between severe periodontitis and cognitive decline. (4) Conclusions: This study, using a representative U.S. adult population aged 65 and over, suggests that lower cognitive performance correlates with higher likelihood of severe periodontitis. ALP enhances the association between severe periodontitis and cognitive decline.

## 1. Introduction

**Figure 1:**
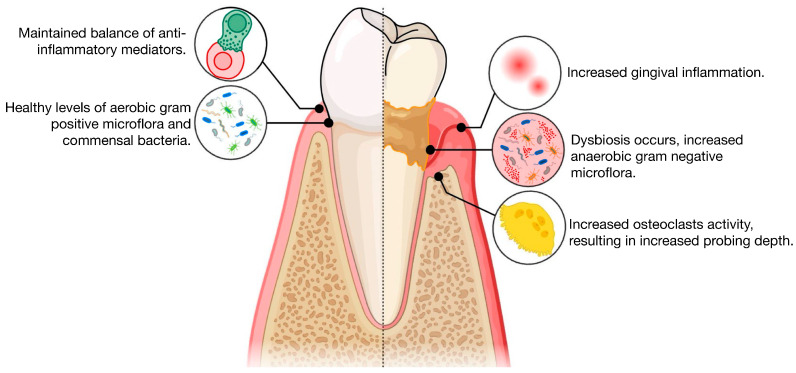
Illustration of the pathogenesis of periodontitis, comparing health with periodontitis.

The decline in cognitive function profoundly affects the daily activities and overall quality of life for those impacted, and it may serve as an early indicator of Alzheimer’s disease (AD), a devastating neurodegenerative condition [1,2]. According to the World Alzheimer Report 2015, it is estimated that by 2030, approximately 74.7 million people will be living with dementia, characterized by cognitive decline, highlighting a significant socioeconomic burden on caregivers and healthcare systems [3]. Recent research has shown that cognitive dysfunction is associated with various inflammatory and metabolic conditions, such as cardiovascular disease and diabetes [4,5,6]. Among these inflammatory conditions, periodontal disease has gained attention for its potential link to cognitive decline [7]. Periodontitis, a chronic low-grade inflammatory condition caused by dental plaque accumulation and exacerbated by systemic inflammatory and genetic factors, not only leads to progressive damage to the supporting structures of the teeth, but also has systemic effects through the release of pro-inflammatory cytokines into the bloodstream [8], Figure 1. The inflammatory response, coupled with bacteria associated with periodontal disease, may contribute to several systemic diseases, including cognitive decline and dementia [9,10]. The transition from predominantly aerobic and Gram-positive bacteria in healthy gingiva to gram-negative bacteria, particularly the “red complex” pathogens in periodontitis, promotes the release of pro-inflammatory cytokines such as interleukin-1β (IL-1β), IL-6, and TNF-α [11]. Additionally, *Porphyromonas gingivalis* (*P. gingivalis*) bacteria, a periodontal pathogen, can directly impact the brain and produce neurotoxic virulence factors that compromise the integrity of blood–brain barrier cells, predisposing individuals to cognitive dysfunction and dementia [12,13].

We hypothesize that there is a positive association between severe periodontitis and the risk of cognitive decline, mediated by inflammatory biomarkers. The aim of this study was to examine the relationship between cognitive function and severe periodontitis in a representative sample of the elderly US population, using data from the National Health and Nutrition Examination Survey (NHANES) for the years 2013 to 2014. Additionally, we investigate whether elevated blood serum levels of alkaline phosphatase (ALP)—an enzyme known to increase with dementia and oral inflammation [14,15]—act as an effect modifier in the relationship between cognitive dysfunction and severe periodontal disease.

## 2. Materials and Methods

### 2.1. Study Population

This study utilized the publicly available National Health and Nutrition Examination Survey (NHANES) cross-sectional survey data from 2013 to 2014, adhering to the Data Use Restrictions set by the National Center for Health Statistics, Centers for Disease Control and Prevention [3]. The inclusion criteria for this study were individuals aged 65 years or older who participated in the NHANES 2013–2014 dataset. The exclusion criteria were individuals who were not part of the NHANES 2013–2014 dataset and were under the age of 65. NHANES is a complex, multistage, stratified, and clustered sampling method to capture data from the civilian, non-institutionalized U.S. population. Eligibility criteria for inclusion in the study required participants to have complete data for the variables of interest. Participants with missing or incomplete records for key variables were excluded.

The NHANES is a comprehensive source of health, disease, and risk factor data representative of the U.S. population, derived from a meticulously designed and conducted study that began in 1999. The NHANES encompasses a range of components, including questionnaires, laboratory tests, and clinical examinations, to measure health outcomes and explanatory variables [4]. This study utilized publicly available data from the National Health and Nutrition Examination Survey (NHANES) and was deemed exempt by the IRB, as it was classified as non-human research.

### 2.2. Periodontal Examination

During the NHANES study from 2013 to 2014, individuals aged 30 years and older without health conditions requiring antibiotic prophylaxis underwent a full-mouth periodontal examination (FMPE). This examination aimed to establish definitive assessments for clinical attachment loss (AL). Direct measurements of the distance between the cemento–enamel junction and the free gingival margin (CEJ-FGM) and the probing depth (PD) were taken at each site to achieve this goal. These measurements were conducted at six specific sites on all teeth except for the third molars. All measurements were rounded to the lower whole millimeter, and clinical AL was calculated based on the recorded measurements. For the purpose of this study, we included only subjects aged 65 years and older who received a periodontal exam. The reason is that only individuals aged 65 and older received the cognitive function assessment, described below as our main exposure variable.

### 2.3. Definition of the Dependent Variable: Severe Periodontitis

The outcome variable of interest was characterized as individuals diagnosed with severe periodontal disease. This condition was identified and coded as individuals exhibiting two or more interproximal sites with at least 6 mm of attachment loss, not on the same tooth, and at least one interproximal site with probing depths of 5 mm or more, defined according to the Centers for Disease Control and Prevention (CDC) and the American Academy of Pediatrics (AAP) case definition [5].

### 2.4. Description of Independent Variable: Cognitive Function 

The primary exposure variable, cognitive function, was evaluated using the Digit Symbol Score (DSS). This score, which ranges from 0 to 100 (with a higher DSS indicating better cognitive health), was derived from a series of tests including digit symbol substitution, animal fluency, recall, intrusion tests, and delayed responses [16,17]. The Digit Symbol Substitution Test (DSST) requires participants to match symbols to numbers based on a provided key within a limited time, assessing processing speed, attention, and working memory. The animal fluency test involves naming as many animals as possible within one minute, evaluating verbal fluency, executive function, and semantic memory. Immediate and delayed recall tests ask participants to memorize and recall a list of words immediately and after a delay, measuring short-term and long-term memory, as well as the ability to encode, store, and retrieve information. Intrusion tests track the incorrect words (intrusions) recalled during the recall tests, providing insights into inhibitory control and the ability to suppress irrelevant information. Lastly, delayed response tests require participants to perform tasks or recall information after a delay, assessing working memory and executive function by measuring the ability to retain and manipulate information over a period. In summary, these assessments collectively measure various cognitive function domains, including memory, processing speed, and executive function, serving as critical indicators of cognitive health in the context of this study. The use of CERAD-based cognitive assessments in this study provides a valuable screening tool, but lacks the precision of clinical diagnostic evaluations for cognitive impairment. This limitation may lead to potential misclassification of cognitive decline, as CERAD scores do not account for factors such as individual variability in baseline cognitive ability or non-neurodegenerative causes of cognitive changes. To overcome this limitation, future studies should incorporate comprehensive clinical diagnostic tools, such as neuroimaging or cerebrospinal fluid biomarkers, alongside validated cognitive tests. These methods would allow for a more accurate classification of cognitive impairment and provide deeper insights into the relationship between cognitive decline and periodontal disease.

### 2.5. The Effect Modifier Variable

Alkaline phosphatase: This is a continuous variable that refers to the measurement of the enzyme ALP in the blood serum of participants. The variable named “LBXSAPSI” in the NHANES laboratory dataset was used to measure ALP serum levels. The sample collection and analysis protocols, following standardized procedures, are detailed on the NHANES website [18]. In brief, samples were collected in appropriate vacutainer tubes to prevent clotting or hemolysis and were immediately processed. Upon arrival at the laboratory, samples underwent centrifugation to separate serum from cellular components. The ALP levels in the serum were quantified using enzymatic methods with results reported in units per liter (IU/L) of serum. This procedure ensured accurate measurement of ALP levels across the NHANES 2013/2014 study cohort [19].

### 2.6. The Potential Confounding Variables

Sex: This was a binary variable, males and females.

Age: Age at screening was used as a continuous variable.

Diabetes status: Participants were asked whether a doctor or health professional had ever diagnosed them with diabetes or pre-diabetes, with responses categorized as follows: ‘no’, ‘yes’, or ‘borderline’ (where ‘borderline’ indicates a pre-diabetic state). This variable was treated as binary (‘0’ for ‘no’ and ‘1’ for ‘yes/borderline’).

Disease status: This variable was dichotomized into two categories (“0” = No and “1” = Yes). A value of “1” was assigned to the category if participants reported a diagnosis of at least one of the following conditions: arthritis, cancer, or coronary heart disease. Conversely, those who were either healthy or did not report any of these chronic diseases were placed in the “0” category.

Smoking status: Smoking status was assessed through questions asked during a household interview [18]. Participants were categorized as either current smokers or those who have never smoked before. Current smokers were defined as individuals who had smoked 100 or more cigarettes in their lifetime and who currently smoke, whether daily or occasionally. Those who have never smoked before were those who indicated they had not smoked 100 cigarettes in their lifetime.

Income level: The income-to-federal poverty level (FPL) ratio was used as a measure of financial status. This metric was segmented into four categories: below 138% FPL, 138% to 199% FPL, 200% to 399% FPL, and ≥400% FPL. The income data used to calculate poverty levels were based on participant responses regarding their household income.

### 2.7. Statistical Method

Chi-square tests were employed to describe the categorical variables, while *t*-tests were used to describe the continuous variables. A weighted multivariate binary logistic regression model was used to test the hypothesis that there is an association between severe periodontitis and the risk of cognitive decline in a nationally representative sample of US elderly, adjusting for age, sex, diabetes status, medical history, smoking status, and poverty level. Additionally, the model explored whether ALP acts as an effect modifier in the relationship between cognitive dysfunction and severe periodontal disease. An interaction variable was created to assess the joint effect of the “cognitive function” variable with the “alkaline phosphatase level” variable, aiming to examine the impact of ALP levels on the association between cognitive function and severe periodontitis. The analysis utilized survey weights and accounted for the complex sampling design of the NHANES 2013/2014 dataset, ensuring that the estimates were representative of the entire US elderly population. Statistical analysis was conducted using STAT 17 software, with the significance level set at 0.05.

## 3. Results

Table 1 presents a descriptive summary of the categorical variables of the population characteristics, comparing individuals with severe periodontal disease with those without it. P values were calculated using the Chi-square test. Among the 4669 individuals in the sample, 329 (7.05%) were diagnosed with severe periodontal disease. Notably, 84.99% of those without severe periodontal disease did not have diabetes. Additionally, 61.04% of individuals with severe periodontitis were current smokers, while 55.6% of those without severe periodontitis were non-smokers. Males were more likely to have severe periodontal disease (69.60%) compared with females (30.40%). Further demographic characteristics are detailed in Table 1.

Table 2 presents a descriptive summary of the continuous variables in comparison with and without severe periodontal disease. Individuals diagnosed with severe periodontitis exhibited lower mean cognitive function scores (42.1 points) compared with those without severe periodontitis (46.3 points), with a *p*-value of 0.006. The serum ALP levels were higher in individuals with severe periodontitis (70.9 IU/L) compared with those without it (67.1 IU/L), with a *p*-value of 0.016.

Table 3 shows the results of the weighted multiple logistic regression model showing the association between severe periodontal disease and cognitive function. For each one-point increase in the cognitive function score, the odds of having severe periodontitis decreased by 2% (OR = 0.98, 95% CI: 0.96–0.99, *p* = 0.018). The interaction term between serum ALP levels and cognitive function also showed a statistically significant association (OR = 1.1, 95% CI: 1.0–1.2, *p* < 0.001), implying that the relationship between cognitive function and periodontal disease may have been modified by serum ALP levels. Age and sex were significant covariates in the model, with individuals aged 65 and older having lower odds of severe periodontal disease (OR = 0.94, 95% CI: 0.90–0.99, *p* = 0.035) and females having lower odds compared with males (OR = 0.32, 95% CI: 0.18–0.57, *p* < 0.001). Diabetes status and smoking status did not show a significant association with severe periodontal disease in this model, with *p*-values of 0.422 and 0.816, respectively. The poverty rate levels also did not show significant associations with severe periodontal disease, although trends suggested lower odds in higher-income brackets (OR = 0.63 for 138%-399% FPL and OR = 0.58 for 400%+ FPL, both *p* = 0.111).

## 4. Discussion

**Figure 2:**
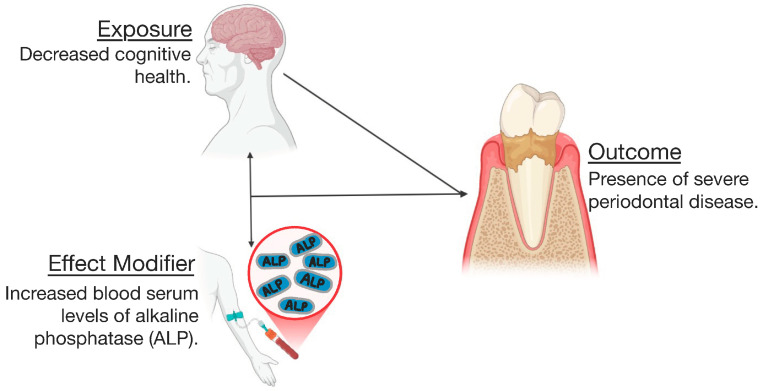
Relationship of exposure, outcome, and effect modifier.

This study demonstrated that better cognitive function is associated with lower risk of severe periodontitis, suggesting that good periodontal health may be protective against cognitive impairment, Figure 2. Additionally, our analysis revealed that serum ALP levels act as an effect modifier in the relationship between cognitive function and severe periodontal disease, Figure 2. In other words, individuals with elevated ALP levels are more likely to show a stronger association between cognitive decline and the risk of developing severe periodontitis. The clinical motivation for evaluating effect modification was to determine whether the impact of cognitive function on severe periodontitis varied according to ALP levels; our study suggests a link between cognitive function, serum ALP levels, and severe periodontal disease [4,20,21,22,23].

ALP is primarily known for its role in bone turnover and mineralization, including resorption processes mediated by osteoclasts, which complicates its potential connection to cognitive function and periodontal disease. Elevated ALP levels often indicate bone metabolism abnormalities, such as osteoporosis and Paget’s disease of bone, rather than cognitive impairment [19]. However, in periodontitis, ALP is upregulated in response to inflammatory signals, contributing to the breakdown of alveolar bone and periodontal ligament [20,21,22,23]. This enzyme’s involvement in the resorption process underscores its potential as an effect modifier in the relationship between periodontitis and systemic outcomes, such as cognitive decline. Recent research has highlighted that ALP, particularly tissue non-specific alkaline phosphatase (TNAP), may play a role in AD through mechanisms involving neuroinflammation and protein aggregation.

The association between periodontitis and cognitive decline observed in this study may be underpinned by several biological mechanisms [10,11,12,24,25,26,27]. Chronic periodontal disease is known to contribute to systemic inflammation, which has been implicated in the pathophysiology of cognitive decline. Immune dysregulation resulting from persistent periodontal inflammation may lead to neuroinflammatory responses that could exacerbate cognitive impairment [28,29,30]. Elevated ALP levels are associated with systemic inflammation and compromised blood–brain barrier integrity, which can lead to neuroinflammation and the accumulation of amyloid-beta (Aβ) and tau proteins in the brain [28]. TNAP dephosphorylates tau, preventing its aggregation under normal conditions. However, in the context of AD, excessive dephosphorylation may enhance tau’s neurotoxic effects via receptor-mediated pathways, potentially contributing to neuronal death [29]. Interactions between ALP, Aβ, and tau proteins may exacerbate neurodegeneration by promoting protein aggregation and inflammatory responses [30]. These findings suggest that ALP could serve as both a biomarker and a therapeutic target in AD.

Despite these insights, our study did not specifically exclude individuals with bone turnover abnormalities or hypophosphatasia, which could influence ALP levels. Thus, interpreting our findings requires caution, as they may also reflect underlying bone turnover issues. Further research is necessary to explore the potential association between cognitive function, ALP levels, and periodontal disease, considering both neurodegenerative and bone health contexts.

The bidirectional relationship between oral health and brain health is an important area of research, highlighting how oral inflammation and an imbalanced oral microbiome can impact brain health [31]. Although the mechanistic pathways linking periodontitis and cognitive decline remain unclear, emerging research sheds light on the roles of systemic inflammation, direct translocation of periodontal pathogens, and vascular reactivity in this relationship [12,25,26,27,32,33]. Studies have detected *Porphyromonas gingivalis* (*P. gingivalis*) DNA or its virulence factors, including lipopolysaccharide and gingipain, in the brains of AD patients, closely associated with AD pathological changes [34,35]. Moreover, mouse models have demonstrated that *P. gingivalis* contributes to neuroinflammation and increased amyloid-beta production, exacerbating Alzheimer’s symptoms in transgenic mice [32]. Similarly, *Fusobacterium nucleatum* (*F. nucleatum*)-induced periodontitis exacerbated Alzheimer’s symptoms in AD-like transgenic mouse models, including increased cognitive impairment, beta-amyloid accumulation, and tau protein phosphorylation in the mouse cerebrum [36].

Although data were not explicitly presented on the oral microbiome’s link to cognitive function due to the dataset’s limitations, a potential connection between an inflammatory biomarker, ALP, severe periodontal disease, and cognitive dysfunction—an early stage of AD. It is crucial to emphasize that ALP’s role in this association may be complex, potentially reflecting underlying bone health while influencing cognitive decline.

Elevated serum ALP levels serve as an effect modifier in the relationship between severe periodontitis and cognitive decline. ALP, an enzyme crucial for biological processes like signal transduction, energy metabolism, and skeletal mineralization, also impacts systemic health conditions [19]. Our findings suggest that ALP intensifies cognitive decline and the risk of developing severe periodontitis. High serum ALP levels have been shown to increase the risk of cognitive impairment [37,38]. Elevated ALP levels in the gingival crevicular fluid (GCF) and saliva of patients with periodontitis indicate its potential as a diagnostic marker for periodontal disease [20,21]. Notably, ALP levels in saliva and GCF decrease following scaling and root planing (SRP), attributed to reduced inflammation and periodontal tissue healing [22,23].

Emerging therapeutic approaches further highlight the potential for mitigating periodontal disease and its systemic effects. Plant-derived compounds, such as myricetin, demonstrate a dual mechanism of action against *P. gingivalis*. Myricetin not only inhibits bacterial growth and the expression of key virulence genes, but also modulates the host’s inflammatory response by suppressing NF-κB activation and reducing pro-inflammatory cytokine secretion in gingival fibroblasts [13,39].

Furthermore, *P. gingivalis* contributes to Alzheimer’s disease (AD) by releasing toxic enzymes, including gingipains, which degrade host proteins, disrupt immune functions, and promote tissue invasion [35,40]. *Treponema denticola* (*T. denticola*) has been implicated in Alzheimer’s disease (AD) pathology, with spirochetes like *T. denticola* identified in the brain tissue of AD patients, suggesting their role in disease progression through chronic infection and inflammation. [41]. Additionally, *F. nucleatum* oral infections have been shown to induce neuroinflammation and memory impairment in animal models, highlighting its potential involvement in neurodegenerative diseases [42]. *Aggregatibacter actinomycetemcomitans* (*Aa*), another periodontal pathogen, produces leukotoxin A (LtxA), which selectively targets human leukocytes, particularly neutrophils. This toxin undermines the host immune response and facilitates bacterial persistence [43]. Notably, the highly leukotoxic JP2 clone of *Aa*, characterized by a promoter region deletion, exhibits elevated LtxA production. Epidemiological studies link this clone to aggressive periodontitis, particularly in adolescents of African descent [44].

Chronic inflammation from periodontal disease also accelerates atherosclerosis and reduces cerebral blood flow, both key risk factors for cognitive impairment [45]. These mechanisms underscore the complex interplay between oral health, systemic inflammation, and neurodegeneration. The impact of microbiological, molecular, and genetic markers on periodontitis progression and its systemic effects, such as cognitive decline, varies significantly with age and geographic location. For instance, younger individuals with localized aggressive periodontitis, such as Localized Stage III Grade C, often exhibit distinct microbial profiles dominated by *Aa* [46], whereas older populations typically display a polymicrobial etiology involving pathogens such as *P. gingivalis* and *T. denticola* [47]. Additionally, genetic polymorphisms, including variations in *IL-1* or *TNF-α* genes, have been shown to influence individual susceptibility to periodontal inflammation and may differ across populations. Geographic variations in diet, environmental exposures, and healthcare access further modulate these microbial and genetic interactions. A deeper understanding of these age- and region-specific differences is essential for developing targeted diagnostic and therapeutic strategies to manage periodontitis and mitigate its systemic effects. Future studies need to include diverse populations and investigate how microbiological, molecular, and genetic profiles contribute to periodontitis and systemic outcomes across various demographics and regions [16,17,48,49]. 

Gingipain inhibitors have shown potential in reducing neuroinflammation and Aβ accumulation, potentially mitigating neurodegenerative processes associated with Alzheimer’s disease (AD) [7,29,34,35,50,51]. The association between cognitive decline and periodontitis may be mediated by systemic inflammation. Periodontal disease induces chronic inflammation, releasing pro-inflammatory cytokines like IL-6, TNF-α, and CRP, which exacerbate neuroinflammation, disrupt the blood–brain barrier, and promote neurodegeneration [28,42].

In periodontitis, an elevated receptor activator of nuclear factor-kappa B ligand (RANKL)/osteoprotegerin (OPG) ratio indicates increased osteoclastic activity, driving bone resorption and alveolar bone loss. [52]. This imbalance plays a critical role in periodontal tissue destruction and aligns with systemic inflammatory pathways, potentially linking periodontal disease to cognitive decline [53,54,55,56]. The involvement of RANKL signaling underscores its importance in disease progression and highlights potential therapeutic targets to mitigate bone destruction. Additionally, the novel role of ALP in the association between cognitive decline and periodontitis warrants further investigation, offering new insights into the interconnected mechanisms underlying these conditions.

Systemic inflammation from periodontitis mediates the association with cognitive decline [45,57]. Individuals with periodontitis exhibited higher levels of serum inflammatory markers, such as C-reactive protein (CRP) and interleukin-6 (IL-6), which are associated with an increased risk of cognitive impairment and AD [51,58,59]. Recently, it was shown that severe periodontitis was linked to increased arterial stiffness, associated with cognitive decline in cardiovascular disease patients [33]. This suggests that vascular changes could mediate the cognitive effects of periodontitis [33]. Furthermore, clinical observations indicate a bidirectional relationship between oral health and brain health, with poor oral hygiene exacerbating systemic inflammation and neuroinflammation in neurodegenerative diseases like AD [31]. All these studies provide evidence of an association between periodontitis and cognitive impairment and AD pathology. Nonetheless, the mechanisms responsible for this association remain unclear and warrant further investigation.

The findings of this study are from the adult population aged 65 years and over in the United States in 2013–2014 (NHANES), utilizing a cross-sectional design. This design limitation restricts the assessment to measuring associations, rather than establishing causation between cognitive function and severe periodontal disease. Additionally, there is a risk of residual confounding factors, such as medical conditions, body weight, and specific hormones that could potentially influence the relationship between cognitive function and severe periodontal disease.

### 4.1. Limitation of the Research

A limitation of this study is the absence of clinical assessment data for cognitive impairment. Without these clinical assessments, there may be limitations in the precision of cognitive impairment diagnosis and classification, which could impact the accuracy of the associations observed in this study, potentially leading to misclassification bias. This limitation highlights the need for future research that includes clinical evaluations to provide a more robust understanding of the relationship between periodontitis and cognitive decline. Additionally, the cross-sectional nature of this study limits causal interpretation of the association between severe periodontitis and cognitive decline.

### 4.2. Future Perspectives

Future studies should focus on longitudinal designs, such as prospective cohort studies or randomized controlled trials, to establish temporal relationships and clarify causal pathways. Further research is needed to uncover the underlying mechanisms of the association between periodontitis and cognitive impairment. This includes collecting oral microbiome data, conducting mechanistic studies, and performing longitudinal analyses. Specifically, future prospective intervention studies and longitudinal data collection would be valuable for elucidating the causal relationship between cognitive function and periodontal health using unbiased measures. In this study, we utilized CDC/AAP case definition classification [5], which primarily focuses on disease severity. The 2019 World Workshop Consensus introduced a standardized classification system for periodontitis that integrates both staging (disease severity and extent) and grading (progression rate and systemic risk factors). While the European classification was suitable for the scope of our analysis, adopting the 2019 Consensus criteria in future studies could provide a more comprehensive understanding of how periodontitis contributes to systemic outcomes, such as cognitive decline.

It is well-established that oral hygiene and diet are critical components of overall health, and their role in cognitive function is no exception. Poor oral hygiene has been linked to periodontal disease, serving as a gateway for systemic inflammation that may impact brain function. A diet and oral hygiene practices that are beneficial to oral and cognitive health may mitigate the risks associated with cognitive decline. Future studies should evaluate the role of these factors alongside the role of ALP and its potential link to bone turnover abnormalities, as well as the influence of nutritional status on the relationship between oral and cognitive health. Exploring how improvements in oral hygiene practices and dietary interventions could modulate the progression of cognitive impairments will provide insights into modifiable risk factors. Such research could advance prevention strategies and improve health outcomes, particularly among older adults who are at an increased risk.

## 5. Conclusions

This study, using a representative sample of the U.S. elderly population aged 65 and older, highlights a significant association between severe periodontitis and low cognitive performance. Alkaline phosphatase (ALP), a marker typically linked to bone turnover, may intensify this relationship, warranting further exploration of its role as a biomarker in cognitive risk profiling. Based on these findings, we recommend incorporating routine oral health assessments and periodontal care into standard older adult health evaluations to reduce the risk of cognitive decline. Public health policies should prioritize expanding access to dental care and oral health education for older adults, particularly in underserved communities, as maintaining periodontal health offers a cost-effective strategy to improve overall health and quality of life. Interdisciplinary collaboration between dentists and physicians will be critical in addressing the shared risks of poor oral and cognitive health.

## Figures and Tables

**Table 1:** Descriptive summary of population characteristics: Comparison with and without severe periodontal disease.

Covariate		No Severe Periodontitis N = 4340 (92.95%)	Severe Periodontitis N = 329 (7.05%)	Total N= 4669	*p*-Value
Sex	male	1987 (45.48%)	229 (69.60%)	2216 (47.46%)	<0.001
	female	2353 (54.22%)	100 (30.40%)	2453 (52.54%)	
Smoking	no	2413 (55.60%)	127 (38.60%)	2540 (54.40%)	<0.001
	yes	1927 (44.40%)	202 (61.04%)	2129 (45.60%)	
Poverty Level	<138%	1343 (30.94%)	129 (39.21%)	1472 (31.53%)	<0.001
	138–399%	1529 (35.23%)	129 (39.21%)	1658 (35.51%)	
	400%+	1468 (33.82%)	71 (21.58%)	1539 (32.96%)	
Diabetes	No	3686 (84.99%)	279 (84.80%)	3965 (84.98%)	0.927
	Yes	651 (15.01%)	50 (15.20%)	701 (15.02%)	
Any disease	No	2372 (54.81%)	196 (59.94%)	2568 (55.17%)	0.072
	Yes	1956 (45.19%)	131 (40.06%)	2087 (44.83%)	

**Table 2:** Descriptive summary of continuous variables: Comparison with and without severe periodontal disease.

Cont. Variable	No Severe Periodontitis Mean (SD)	Severe Periodontitis Mean (SD)	*p*-Value
Cognitive Function	46.3 (17.3)	42.1 (15.1)	0.006
Serum Alkaline Phosphatase	67.1 (27.4)	70.9 (23.0)	0.016
Age in years	53.8 (14.9)	56.9 (11.9)	<0.001

**Table 3:** Weighted multiple logistic regression model: The association between severe periodontal disease and cognitive function in the United States, 2013–2014.

Covariate		Composite			
		Odds Ratio	Confidence Interval		*p* Value
			Lower	Upper	
Cognitive Function		0.98	0.96	0.99	0.018
Alkaline Phosphatase Cognition (interaction term)		1.1	1.0	1.2	<0.001
Age		0.94	0.90	0.99	0.035
Sex (male is the reference)		0.32	0.18	0.57	<0.001
Diabetes Status (no diabetes is the reference)		1.3	0.66	2.6	0.422
Smoking Status (no smoking is the reference)		0.93	0.53	1.6	0.816
Income (below 138% FPL is the reference)	138–399% FPL	0.63	0.35	1.1	0.111
	400% FPL	0.58	0.30	1.1	0.111

## Data Availability

The data supporting the findings of this study can be made available by the corresponding author upon reasonable request. Due to privacy considerations, data sharing will be subject to appropriate safeguards and approvals. For further details, inquiries can be directed to the corresponding author.
